# Intranasal Mesenchymal Stem Cell Treatment for Neonatal Brain Damage: Long-Term Cognitive and Sensorimotor Improvement

**DOI:** 10.1371/journal.pone.0051253

**Published:** 2013-01-03

**Authors:** Vanessa Donega, Cindy T. J. van Velthoven, Cora H. Nijboer, Frank van Bel, Martien J. H. Kas, Annemieke Kavelaars, Cobi J. Heijnen

**Affiliations:** 1 Laboratory of Neuroimmunology and Developmental Origins of Disease, University Medical Centre Utrecht, Utrecht, The Netherlands; 2 Department of Neonatology, University Medical Center Utrecht, Utrecht, The Netherlands; 3 Rudolf Magnus Institute of Neuroscience, Department of Neuroscience and Pharmacology, University Medical Center Utrecht, Utrecht, The Netherlands; 4 Department of Symptom Research, MD Anderson Cancer Center, Houston, Texas, United States of America; Hôpital Robert Debré, France

## Abstract

Mesenchymal stem cell (MSC) administration via the intranasal route could become an effective therapy to treat neonatal hypoxic-ischemic (HI) brain damage. We analyzed long-term effects of intranasal MSC treatment on lesion size, sensorimotor and cognitive behavior, and determined the therapeutic window and dose response relationships. Furthermore, the appearance of MSCs at the lesion site in relation to the therapeutic window was examined. Nine-day-old mice were subjected to unilateral carotid artery occlusion and hypoxia. MSCs were administered intranasally at 3, 10 or 17 days after hypoxia-ischemia (HI). Motor, cognitive and histological outcome was investigated. PKH-26 labeled cells were used to localize MSCs in the brain. We identified 0.5×10^6^ MSCs as the minimal effective dose with a therapeutic window of at least 10 days but less than 17 days post-HI. A single dose was sufficient for a marked beneficial effect. MSCs reach the lesion site within 24 h when given 3 or 10 days after injury. However, no MSCs were detected in the lesion when administered 17 days following HI. We also show for the first time that intranasal MSC treatment after HI improves cognitive function. Improvement of sensorimotor function and histological outcome was maintained until at least 9 weeks post-HI. The capacity of MSCs to reach the lesion site within 24 h after intranasal administration at 10 days but not at 17 days post-HI indicates a therapeutic window of at least 10 days. Our data strongly indicate that intranasal MSC treatment may become a promising non-invasive therapeutic tool to effectively reduce neonatal encephalopathy.

## Introduction

Neonatal encephalopathy due to perinatal hypoxia-ischemia (HI) remains a significant cause of neonatal mortality and long-term neurological deficits such as cerebral palsy, mental retardation and seizures in babies born at term [1]–[6]. Presently, the only available treatment, hypothermia, has limited beneficial effects and is only effective in mildly-affected children born at term [7], [8]. Moreover, hypothermia has a narrow therapeutic window of 6 hours. Hence, there is an urgent need to develop therapeutic strategies with a longer therapeutic window.

One emerging strategy with therapeutic potential is mesenchymal stem cell (MSC) treatment. A growing number of studies in rodent models show that MSC treatment significantly improves motor outcome and reduces lesion volume after neonatal brain injury [9]–[18]. Currently, in most studies, MSCs are administered intracranially, which has serious disadvantages for clinical application. In a previous study, we explored the potential of intranasal MSC administration in a mouse model of neonatal HI brain damage. Our results showed that intranasal MSC treatment improved sensorimotor behaviour and decreased lesion volume 4 weeks after HI, suggesting a therapeutic potential [16].

Neonatal encephalopathy in humans is often associated with cognitive impairment [2], [3], [5]. Therefore, we investigated for the first time whether intranasal MSC treatment after neonatal HI brain damage restores cognitive function and sensorimotor function 8 weeks after HI. We also determined the dose response relationships and therapeutic window of intranasal treatment in the HI mouse model. Moreover, we explored the early presence of MSCs at the lesion site in relation to the therapeutic window.

## Materials and Methods

### Ethics statement

Experiments were performed according to the international guidelines and approved by the Experimental Animal Committee Utrecht (University Utrecht, Utrecht, Netherlands).

### Animals

Unilateral HI cerebral damage was induced in 9-day old C57Bl/6 mice by permanent occlusion of the right common carotid artery under isoflurane anesthesia followed by 45 min 10% oxygen at 35°C [14]. Our HI induction procedure has a 10% death rate. Sham-controls underwent anesthesia and incision only. Pups from at least five litters were randomly assigned to experimental groups. Analyses were performed in a blinded set-up.

Mesenchymal stem cells from C57/bl6 mice were purchased from Invitrogen (GIBCO mouse C57Bl/6 MSCs, Life Technologies, UK) and cultured according to the manufacturer's instructions. Characterization of cell specific antigens was performed in a previous study from our group [16]. 3 µl of hyaluronidase in PBS (100 U, Sigma-Aldrich, St. Louis, MO) was administered to each nostril. Thirty minutes later animals received 3 µl MSCs or 3 µl PBS (vehicle) twice in each nostril.

### Sensorimotor function

Unilateral sensorimotor deficits were evaluated in the cylinder rearing test (CRT). Weight-bearing left (impaired), right (unimpaired) or both paw(s) contacting the wall during full rear were counted. Paw preference was calculated as ((right−left)/(right+left+both))×100%.

### Cognitive function

To assess cognitive function, we used the social discrimination test as described [19]. After 10 minutes of habituation to the test environment, the test mouse was allowed to explore a novel conspecific of the same gender (Mouse 1), which is placed in a wire cage for 10 minutes. Five minutes later, the test mouse is exposed to a novel mouse (Mouse 2), which is placed in the empty wire cage while Mouse 1 remains in the other side chamber. This session is repeated 5 min and 3 hours later with a novel unfamiliar mouse (Mouse 3 and Mouse 4) and the now-familiar Mouse 2. Time spent interacting with the familiar or unfamiliar conspecific was scored. Percent time spent with the novel mouse was calculated as ((interaction time novel mouse)/(total interaction time)).

### Histology

Coronal paraffin sections (8 µm) of paraformaldehyde (PFA)-fixed brains were incubated with mouse-anti-myelin basic protein (MBP) (Sternberger Monoclonals, Lutherville, MD,) or mouse-anti-microtubuli-associated protein 2 (MAP2) (Sigma-Aldrich) followed by biotinylated horse-anti-mouse antibody (Vector Laboratories, Burliname, CA). Binding was visualized with Vectastain ABC kit (Vector Laboratories) and diaminobenzamidine. Ipsilateral MAP2 and MBP area loss were determined on sections −1.85 mm from bregma [20]. MBP and MAP2 staining were quantified using ImageJ software (http://rsb.info.nih.gov/ij/) and Adobe Photoshop CS5, respectively.

### MSC tracking

MSCs were labeled with PKH-26 Red fluorescent cell linker kit (Sigma-Aldrich) and administered intranasally. 24 hours after MSC treatment brains were fixed in 4% PFA. Coronal cryosections (8 µm) were stained with DAPI. Fluorescent images were captured using a EMCCD camera (Leica Microsystems, Benelux) and Softworx software (Applied Precision, Washington).

### Statistical analysis

Data were analyzed using (repeated measures) one-way ANOVA followed by Bonferroni post-tests. Significance for social discrimination was analyzed with the one sample t-test. p<0.05 was considered statistically significant. Data are presented as mean ± SEM.

## Results

### Dose of intranasally administered MSC

**Figure 1:**
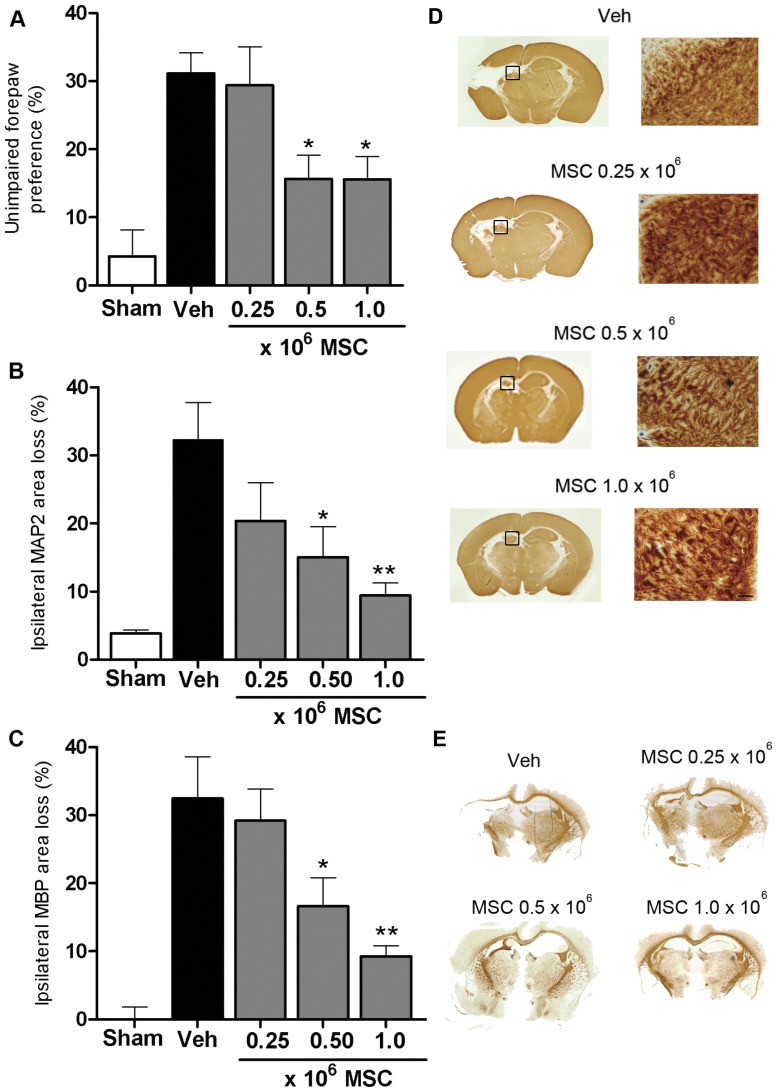
Dose effect of MSCs on motor performance and lesion volume at 35 days post-HI. Mice received 0.25×10^6^, 0.5×10^6^, 1×10^6^ MSCs or Vehicle (Veh) treatment at 10 days after induction of HI. (A) Paw preference to use the unimpaired forepaw in the cylinder rearing test (CRT) was assessed at 5 weeks post-HI. Sham-operated littermates (Sham) were used as controls. Quantification of ipsilateral MAP2 (B) and MBP(C) area loss measured as 1- (ipsi-/contralateral MAP2- or MBP-positive area) at 5 weeks post-HI. (D) Representative sections of MAP2 loss. Insets show higher magnification of corresponding MAP2 sections. Scale bar = 100 µm. (E) Representative sections of MBP area loss. Data represent mean ± SEM. Sham n = 8; Veh n = 10; 0.25×10^6^ MSC n = 11; 0.5×10^6^ MSC n = 10; 1×10^6^ MSC n = 13. *p<0.05; **p<0.01 vs Veh. Data presented in this figure are results from pooled experiments out of 8 different litters. Treatment groups were randomly distributed between litters.

To determine the MSC dose response relationship, we administered 0.25×10^6^, 0.5×10^6^, 1×10^6^ MSCs or vehicle intranasally at 10 days post-HI. Exposure to HI markedly impaired sensorimotor performance in the CRT (Figure 1A).

The doses of 0.5×10^6^ and 1×10^6^ MSCs significantly improved sensorimotor function as determined 3, 4 and 5 weeks post-HI (data shown for 5 weeks; Figure 1A). The beneficial effect of MSC treatment on sensorimotor function was lost when the dose was reduced to 0.25×10^6^ MSCs.

We analyzed MAP2 loss as a measure of gray matter damage and MBP loss as a parameter of white matter loss at 5 weeks post-insult [14]. HI-mice receiving 0.5×10^6^ or 1×10^6^ MSCs on day 10 showed a substantial decrease in MAP2 loss (Figure 1B) and MBP loss (Figure 1C). When a lower dose of 0.25×10^6^ MSCs was used, we no longer observed a beneficial effect on MAP2 or MBP loss.

### Therapeutic window

**Figure 2:**
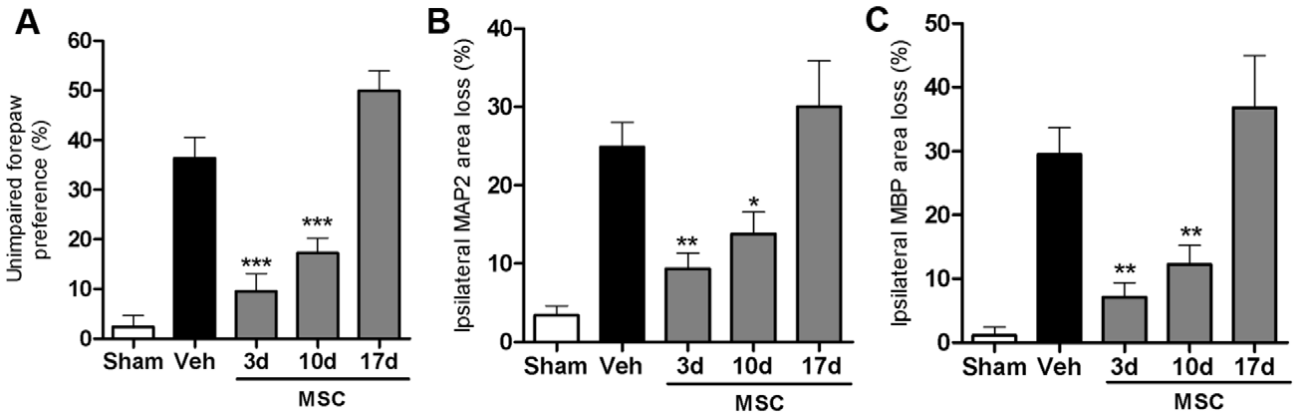
Therapeutic window for MSC treatment. Mice received 0.5×10^6^ MSCs or Veh at 3, 10 or 17 days post-HI. Because no significant difference was found between Veh groups treated at different time points we pooled all animals into one group. (A) Unimpaired forepaw preference in the CRT at 5 weeks post-insult. Quantification of ipsilateral MAP2 (B) and MBP (C) area loss measured as 1- (ipsi-/contralateral MAP2- or MBP-positive area) at 5 weeks post-insult. Insets show representative examples of MAP2 or MBP staining. Data represent mean ± SEM. Sham n = 8; Veh n = 19; MSC 3 d n = 12; MSC 10 d n = 17; MSC 17 d n = 9; *p<0.05; **p<0.01; ***p<0.001 vs. Veh. Data presented in this figure are results from pooled experiments out of 12 different litters. Treatment groups were randomly distributed between litters.

To determine the therapeutic window of intranasal MSC treatment, we administered 0.5×10^6^ MSCs at 3, 10 or 17 days post-HI and assessed sensorimotor performance in the CRT. Our data show that MSC treatment given 3 or 10 days post-insult is effective in improving motor behavior significantly. However, when we postponed MSC treatment to 17 days after HI, we no longer observed improvement of sensorimotor function as analyzed 5 weeks post-insult (Figure 2A).

With respect to lesion volume, MSC treatment at 3 or 10 days, but not 17 days after HI significantly reduced MAP2 and MBP loss (Figure 2B and C).

### Effect of two MSC dosages

**Figure 3:**
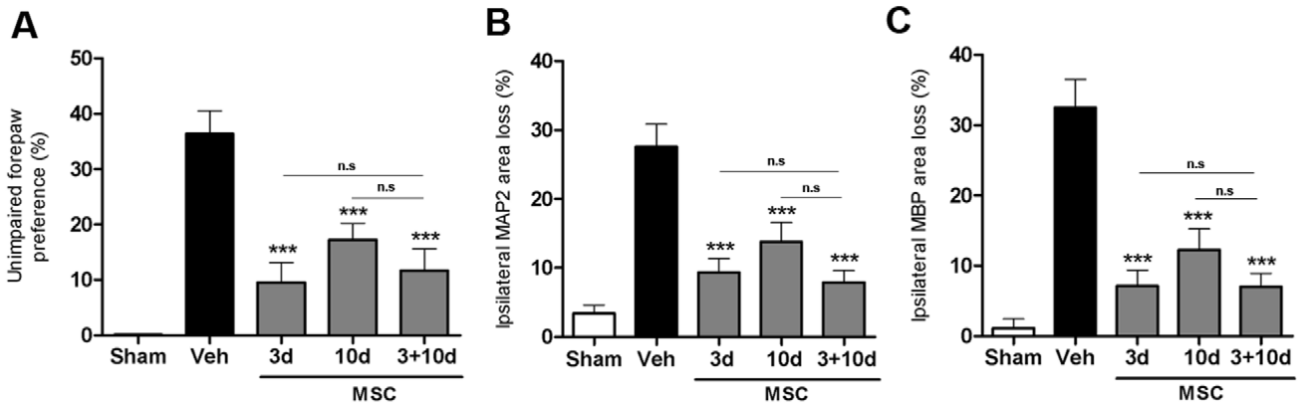
Effect of two MSC treatments on sensorimotor function and lesion size. Mice received 0.5×10^6^ MSCs or Veh at 3, 10 or 3+10 days post-insult. Because no significant difference was found between Veh groups treated at different time points we pooled all animals into one group. (A) Unimpaired forepaw preference in the CRT at 5 weeks post-HI. Quantification of ipsilateral MAP2 (B) and MBP (C) area loss measured as 1- (ipsi-/contralateral MAP2- or MBP-positive area) at 5 weeks post-HI. Insets show representative examples of MAP2 or MBP staining. Data represent mean ± SEM. Sham n = 7; Veh n = 19; MSC 3+10 d n = 13; MSC 10+17 d n = 11; MSC 10 d n = 17. ***p<0.001 vs. Veh., n.s. = non-significant. Data presented in this figure are results from pooled experiments out of 14 different litters. Treatment groups were randomly distributed between litters.

**Figure 4:**
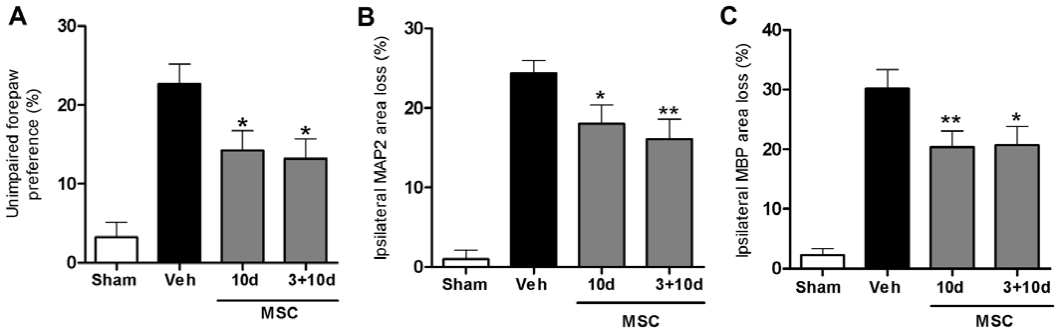
Long-term effect of MSC treatment on sensorimotor function and lesion volume. Mice received 0.5×10^6^ MSCs or Veh at 3, 10 or 3+10 days post-HI. Because no significant difference was found between Veh groups treated at different time points we pooled all animals into one group. (A) Unimpaired forepaw preference in the CRT at 8 weeks post-HI. Quantification of ipsilateral MAP2 (B) and MBP (C) area loss measured as 1- (ipsi-/contralateral MAP2- or MBP-positive area). Insets show representative examples of MAP2 or MBP staining at 9 weeks post-insult. Data represent mean ± SEM. Sham n = 23; Veh n = 23; MSC 10 d n = 23; MSC 3+10 d n = 12. *p<0.05; **p<0.01 vs. Veh. Data presented in this figure are results from pooled experiments out of 13 different litters. Treatment groups were randomly distributed between litters.

Previous results from our group showed that two intracranial MSC injections at 3+10 days compared to a single injection 3 days post-HI, further improved motor performance and decreased neuronal and white matter loss [14]. The data in Figure 3A show that one intranasal dose at either 3 or 10 days is as effective in restoring sensorimotor function as two intranasal doses at 3+10 days post-insult. Histological data on MAP2 or MBP staining confirmed the functional CRT analyses, showing that one single intranasal dose at 3 or 10 days is as effective as two doses at 3+10 days analyzed at 5 weeks post-HI (Figure 3B and C). Furthermore, the positive effects of either one or two intranasal doses of MSCs on sensorimotor and histological outcome can still be observed at respectively 8 and 9 weeks after HI, and no additive effect of two doses was observed at this time point either (Figure 4).

### Effect of MSC treatment on cognitive function

To determine whether MSC treatment improves HI-induced cognitive impairment, we used the social discrimination test at 7 weeks post-HI. This test uses the preference for social novelty as a measure of cognitive function *i.e.* the ability to discriminate between known or unknown conspecific.

**Figure 5:**
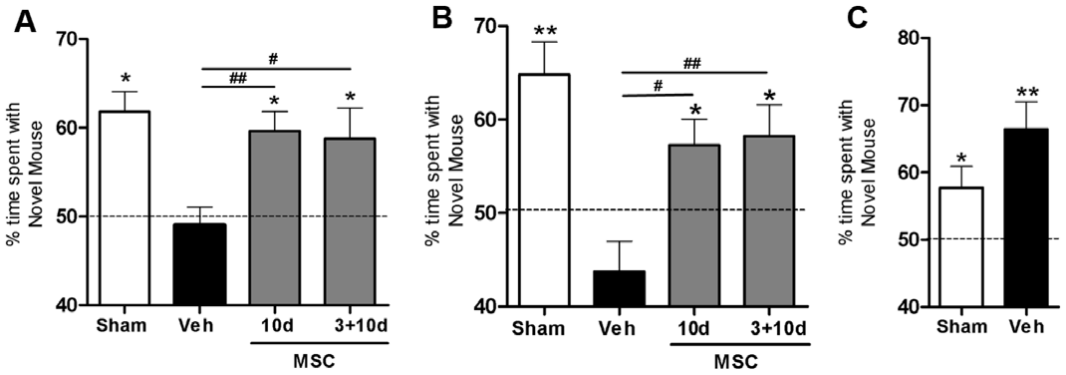
Effect of MSC treatment on cognitive behavior. Mice received 0.5×10^6^ MSCs or Veh at 10 or 3+10 days post-HI. Because no significant difference was found between Veh groups treated at different time points we pooled all animals into one group. Animals were tested for cognitive function using the social discrimination test at 7 weeks post-HI. Preference for novel conspecific is expressed as exploratory ratio. The total social interaction time did not differ between groups. (A) Preference for novel mouse after a 5 min interval. (B) Preference for novel mouse after a 3 hour interval. (C)Training session to measure preference for social novelty as an indication for social avoidance. Sham n = 23; Veh n = 23; MSC 10 d n = 23; MSC 3+10 d n = 12. *p<0.05; **p<0.01 in relation to 50% (no discrimination); ^#^p<0.05; ^##^p<0.01 vs. Veh. Data presented in this figure are results from pooled experiments out of 13 different litters. Treatment groups were randomly distributed between litters.

As anticipated, sham-control mice showed preference for the novel mouse at 5 min and 3 h after the training session (Figure 5A and B). Vehicle-treated HI mice lost the capability to discriminate between the novel and familiar mouse at both time points. Interestingly, performance in the social discrimination test normalized in the groups treated with MSCs at 10 days or 3+10 days following HI. During the training session we did not observe group differences in social interaction times (Figure 5C).

### MSC migration towards lesion site

**Figure 6:**
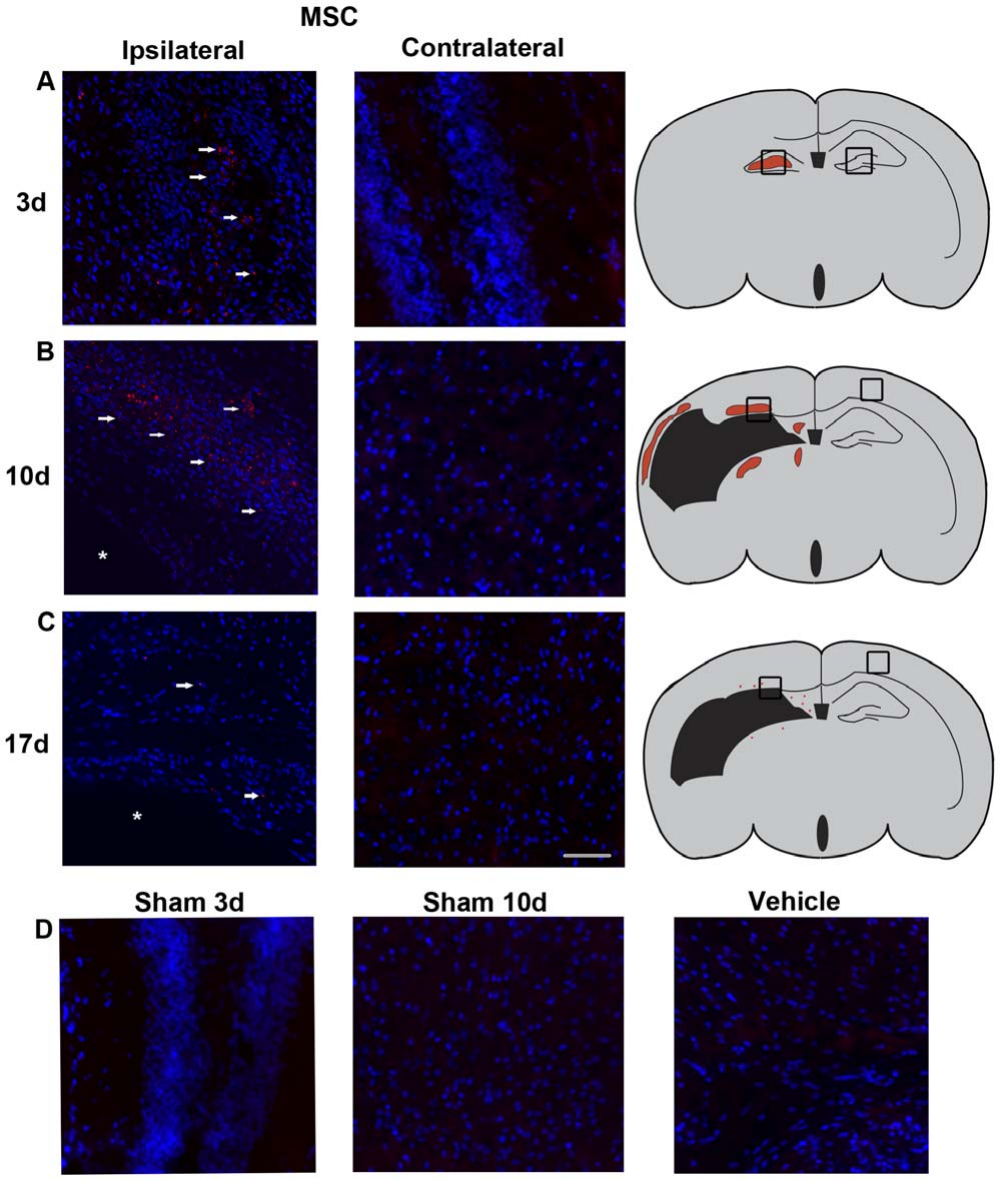
Presence of MSCs in the brain. PKH-26 labeled 1.0×10^6^ MSCs were administered intranasally at 3, 10 and 17 days post-HI. Because no significant difference was found between Veh groups treated at different time points we pooled all animals into one group. (A, B, C) Notice the severe HI-induced damage, as the layer structure of the ipsilateral cortex and hippocampus are lost. (A) MSCs (red) in the ipsilateral hippocampus (see arrow heads) 24 h after administration at 3 days post-HI. (B) MSCs (see arrow heads) in the ipsilateral damaged cortex 24 h after administration at 10 days post-HI. (C) Lack of MSCs (see arrow heads) in the ipsilateral cortical areas surrounding the lesion site when MSCs are given at 17 days post-HI. Contralateral pictures depict hippocampal area (in A) and cortical area (in B and C). (D) Control groups showing lack of MSCs in the hippocampal area and cortical area at 3 and 10 days, respectively, after MSC administration in sham-operated animals and HI-Vehicle treated brain without MSC treatment. Asterisk = lesion site. Blue = Dapi staining. Scale bar 50 µm. Data presented in this figure are results from pooled experiments out of 10 different litters. Treatment groups were randomly distributed between litters.

To determine whether MSCs migrate from the nose to the lesion site, we used PKH-26 labeled MSCs and analyzed brain sections 24 h after intranasal administration. PKH-26+ MSCs administered 3 days post-HI are present predominantly in the damaged hippocampus (Figure 6A; ipsilateral).

MSCs given 10 days after HI localize in the area surrounding the lesion site. At this time point, the entire hippocampus was lost and PKH-26+ cells were now present in cortical layers 5 and 6 (Figure 6B; ipsilateral) and in the dorsal and epithalamic regions (data not shown). Interestingly, hardly any MSCs could be detected in the brain when given 17 days post-HI (Figure 6C; ipsilateral).

No labeled MSCs were present in the contralateral hemisphere, although MSCs were given in both nostrils (Figure 6A to C). In addition, no PKH-26+ cells were observed in brains from sham-operated mice (Figure 6D; sham 3 d and 10 d). Furthermore, we found no evidence for tissue autofluorescence after HI either 3 or 10 days following the insult (Figure 6D; vehicle).

## Discussion

In the present study we show that intranasally administered MSCs reach the brain within just 24 h after intranasal administration. Interestingly, the MSCs migrate specifically towards the lesion site. We determined the dose response relationship of MSC administration and demonstrate that one single intranasal MSC administration is sufficient to induce maximal improvement of functional outcome and reduction in brain damage. A single intranasal dose of MSC has long-lasting effects on motor, cognitive and histological outcome up to 9 weeks following HI injury. Moreover, we are the first to show that intranasal MSC treatment also improves cognitive function, which holds significant clinical relevance.

To determine whether the beneficial effects of intranasal MSC application are associated with migration of MSCs from the nasal mucosa to the lesion site, we used the cell tracking dye PKH-26. In contrast to other commonly used cell tracking dyes, PKH-26 does not have any adverse effects on cell proliferation or survival. Furthermore, no significant cell to cell transfer or dye leakage from cells was measured [21]. No PKH-26+ MSCs were observed at the hippocampal level of sham-operated mice treated at 3 or 10 days post-HI. Interestingly, we clearly detected PKH-26 labeled MSCs at the HI lesion site, but not in the contralateral hemisphere of mice subjected to HI that were treated with MSCs via both the ipsilateral and contralateral nostril. MSCs given at 3 days post-HI migrate specifically to the damaged hippocampus. At 10 days the damage has evolved into an extensive lesion site, including the entire hippocampus and part of the cortex. When MSCs are administered intranasally at 10 days following injury, 24 h later MSCs are located in the remaining regions of the cortex and thalamus. In contrast, when MSCs were administered at 17 days post-HI, only a sporadic PKH-26+ MSC was detected in the lesion, although there is still substantial injury at 17 days post-insult. Notably, MSC treatment at this time point did not have any effect on lesion size nor on sensorimotor function. Combined, these findings indicate that the loss of effect of MSCs given at 17 days after the insult will be due to reduced migration of MSC to the lesion site when cells are given at this time point.

The social discrimination test was used to assess cognitive performance following HI. This test was chosen as it is a very sensitive measure for short-term and long-term recognition memory function [19]. This type of memory describes the ability to discriminate between familiar and unfamiliar stimuli. The social discrimination test exploits the natural interest of an adult mouse to explore an unknown conspecific over a known conspecific. The molecular mechanisms and neuro-anatomical structures underlying this behavioural paradigm remain elusive. Studies have shown that performance on the social discrimination test requires functional olfactory bulb, bed nucleus of the stria terminalis and amygdalo-hippocampal network [22], [23]. The hippocampus is known to be important in memory consolidation, which is essential for long-term memory storage in other cortical areas [24]. We are the first to establish that intranasal MSC treatment not only improves sensorimotor but also cognitive behavior.

The exact mechanisms underlying MSC-induced improvement of short-term memory after neonatal HI brain damage have yet to be clarified. HI injury leads to extensive unilateral loss in cortical and hippocampal areas, which explains the inability of vehicle-treated group to form and/or store new information. Intranasal MSC treatment significantly reduces loss of cortical and hippocampal areas, which are both important in memory formation and storage.

Our present and previous findings [14] indicate that there is no further increase in lesion size after day 10 post-HI. Therefore, we propose that the major effect of MSC treatment is not mediated by inhibition of injurious processes, but rather by stimulating repair. We indeed have evidence from earlier studies in which we applied MSC intracranially that formation of new cells and differentiation of these new cells into neurons and oligodendrocytes is promoted by MSC treatment [14], [15]. Moreover, we showed that only a very small proportion of cells of donor origin survived in the brain [16]. Therefore, it is highly likely that MSC treatment restores cognitive as well as motor circuitries in the brain through stimulation of endogenous regenerative processes.. However, it may well be possible that inhibition of injurious processes contributes to the observed beneficial effects of MSC treatment. If so, this will especially be the case when MSC are administered early after the insult. In our experiments this is 3 days after the insult as at this time point the damage has not yet fully developed.

We also propose that beneficial effects of MSC transplantation are cell-specific, because intracranial fibroblast (3T3) administration did not affect performance in the CRT as well as on lesion size (unpublished observations). Although we now studied the effect of intranasal administration, we anticipate that the same cellular specificity will apply for intranasal administration, give an effect on repair, it should anyhow occur when cells are given directly at the lesion site.

In preparation for clinical translation of our finding that nasally administered MSCs improve cognitive as well as sensorimotor outcome and reduce lesion size, we investigated the therapeutic window, the treatment dose and frequency. We report here that sensorimotor function improves and lesion size reduces significantly when 0.5×10^6^ cells are administered via the nasal route. Decreasing the dose to 0.25×10^6^ MSCs had no significant effect on either the CRT performance or MAP2 and MBP outcome, while increasing the dose to 1.0×10^6^ did not further improve sensorimotor function or reduce lesion size. Thus, a minimum of 0.5×10^6^ MSCs per mouse is required to have a long-lasting beneficial effect on behavior and lesion size in our model of HI-induced brain damage.

In the present study, we demonstrate that multiple doses at 3+10 days via the intranasal route did not have an additional effect on sensorimotor performance and brain damage compared to a single dose. This finding is clinically important as it would mean that only one intranasal dose of MSCs will be sufficient for optimal therapeutic benefit in the neonate. In contrast, we showed earlier that when MSCs are administered intracranially, two gifts of MSC have a stronger beneficial effect on outcome than one gift. One possible explanation for the finding that one intranasal MSC treatment already provides optimal effects is that intranasally administered MSCs may perform better as migration from the nasal mucosa towards the lesion may allow adaptation to the detrimental milieu in the brain. In contrast, intracranially administered cells were directly injected adjacent to the lesion size, consisting of an apoptotic/inflammatory milieu which may partially impair the functionality of MSCs.

## Conclusions

In conclusion, this study shows that intranasal MSC treatment has a wide therapeutic window, leads to long-term improvement in sensorimotor and cognitive function and decreases gray and white matter damage after HI. MSC migrate specifically to the site of injury, despite contralateral administration. Our results clearly establish that intranasal MSC treatment has the potential to become a novel therapeutic strategy for neonatal encephalopathy.

## References

[1] Dammann O, Ferriero D, Gressens P (2011) Neonatal encephalopathy or hypoxic ischemic encephalopathy? Appropriate terminology matters. Pediatr Res 70: 1–2. 21654279 10.1203/PDR.0b013e318223f38d

[2] De Haan M, Wyatt JS, Roth S, Vargha-Khadem F, Gadian D, et al (2006) Brain and cognitive-behavioural development after asphyxia at term birth. Dev Sci 9: 350–358. 16764608 10.1111/j.1467-7687.2006.00499.x

[3] Ferriero DM (2004) Neonatal brain injury. N Engl J Med 351: 1985–1995. 15525724 10.1056/NEJMra041996

[4] Graham EM, Ruis KA, Hartman AL, Northington FJ, Fox HE (2008) A systematic review of the role of intrapartum hypoxia-ischemia in the causation of neonatal encephalopathy. Am J Obstet Gynecol 199: 587–595. 19084096 10.1016/j.ajog.2008.06.094

[5] van Handel M, Swaab H, de Vries LS, Jongmans MJ (2007) Long-term cognitive and behavioural consequences of neonatal encephalopathy following perinatal asphyxia: a review. Eur J Pediatr 166: 645–654. 17426984 10.1007/s00431-007-0437-8 PMC1914268

[6] Volpe JJ (2001) Perinatal brain injury: from pathogenesis to neuroprotection. Ment Retard Dev Disabil Res Rev 7: 56–64. 11241883 10.1002/1098-2779(200102)7:1<56::AID-MRDD1008>3.0.CO;2-A

[7] Edwards AD, Brocklehurst P, Gunn AJ, Halliday H, Juszczak E, et al (2010) Neurological outcomes at 18 months of age after moderate hypothermia for perinatal hypoxic ischaemic encephalopathy: synthesis and meta-analysis of trial data. BMJ 340: c363–370. 20144981 10.1136/bmj.c363 PMC2819259

[8] Azzopardi DV, Strohm B, Edwards AD, Dyet L, Halliday HL, et al (2009) Moderate hypothermia to treat perinatal asphyxial encephalopathy. N Engl J Med 361: 1349–1358. 19797281 10.1056/NEJMoa0900854

[9] Borlongan CV, Weiss MD (2011) Baby STEPS: A giant leap for cell therapy in neonatal brain injury. Pediatr Res 70: 3–9. 21659957 10.1203/PDR.0b013e31821d0d00 PMC3117246

[10] Lee JA, Kim BI, Jo CH, Choi CW, Kim EK, et al (2010) Mesenchymal stem-cell transplantation for hypoxic-ischemic brain injury in neonatal rat model. Pediatr Res 67: 42–46. 19745781 10.1203/PDR.0b013e3181bf594b

[11] Pimentel-Coelho PM, Mendez-Otero R (2010) Cell therapy for neonatal hypoxic-ischemic encephalopathy. Stem Cells Dev 19: 299–310. 19916801 10.1089/scd.2009.0403

[12] Titomanlio L, Kavelaars A, Dalous J, Heijnen C, Baud O, et al (2011) Stem cell therapy for neonatal brain injury: perspectives and challenges. Ann of Neurol 70: 698–712. 22162055 10.1002/ana.22518

[13] van Velthoven CT, Kavelaars A, Heijnen CJ (2012) Mesenchymal stem cells as a treatment for neonatal ischemic brain damage. Pediatr Res 71: 474–481. 22430383 10.1038/pr.2011.64

[14] van Velthoven CT, Kavelaars A, van Bel F, Heijnen CJ (2010) Mesenchymal stem cell treatment after neonatal hypoxic-ischemic brain injury improves behavioral outcome and induces neuronal and oligodendrocyte regeneration. Brain Behav Immun 24: 387–393. 19883750 10.1016/j.bbi.2009.10.017

[15] van Velthoven CT, Kavelaars A, van Bel F, Heijnen CJ (2010) Repeated mesenchymal stem cell treatment after neonatal hypoxia-ischemia has distinct effects on formation and maturation of new neurons and oligodendrocytes leading to restoration of damage, corticospinal motor tract activity, and sensorimotor function. J Neurosci 30: 9603–9611. 20631189 10.1523/JNEUROSCI.1835-10.2010 PMC6632441

[16] van Velthoven CT, Kavelaars A, van Bel F, Heijnen CJ (2010) Nasal administration of stem cells: a promising novel route to treat neonatal ischemic brain damage. Pediatr Res 68: 419–422. 20639794 10.1203/PDR.0b013e3181f1c289

[17] Yasuhara T, Matsukawa N, Yu G, Xu L, Mays RW, et al (2006) Behavioral and histological characterization of intrahippocampal grafts of human bone marrow-derived multipotent progenitor cells in neonatal rats with hypoxic-ischemic injury. Cell Transplant 15: 231–238. 16719058 10.3727/000000006783982034

[18] Yasuhara T, Hara K, Maki M, Mays RW, Deans RJ, et al (2008) Intravenous grafts recapitulate the neurorestoration afforded by intracerebrally delivered multipotent adult progenitor cells in neonatal hypoxic-ischemic rats. J Cereb Blood Flow Metab 28: 1804–1810. 18594556 10.1038/jcbfm.2008.68 PMC2587070

[19] Nadler JJ, Moy SS, Dold G, Trang D, Simmons N, et al (2004) Automated apparatus for quantitation of social approach behaviors in mice. Genes Brain Behav 3: 303–314. 15344923 10.1111/j.1601-183X.2004.00071.x

[20] Nijboer CH, Kavelaars A, Vroon A, Groenendaal F, van Bel F, et al (2008) Low endogenous G-protein-coupled receptor kinase 2 sensitizes the immature brain to hypoxia-ischemia-induced gray and white matter damage. J Neurosci 28: 3324–3332. 18367599 10.1523/JNEUROSCI.4769-07.2008 PMC6670601

[21] Ashley DM, Bol SJ, Waugh C, Kannourakis G (1993) A novel approach to the measurement of different in vitro leukaemic cell growth parameters: The use of PKH GL fluorescent probes. Leuk Res 17: 873–882. 7692186 10.1016/0145-2126(93)90153-c

[22] Auger CJ, Vanzo RJ (2005) Progesterone Treatment of Adult Male Rats Suppresses Arginine Vasopressin Expression in the Bed Nucleus of the Stria Terminalis and the Centromedial Amygdala. J of Neuroendoc 18: 187–194. 10.1111/j.1365-2826.2005.01400.x 16454802

[23] Ferguson JN, Young LJ, Insel TR (2002) The neuroendocrine basis of social recognition. Front Neuroendocrinol 23: 200–224. 11950245 10.1006/frne.2002.0229

[24] Morgado-Bernal I (2011) Learning and memory consolidation: Linking molecular and behavioural data. Neurosci 176: 12–19. 10.1016/j.neuroscience.2010.12.056 21215299

